# An Energy-Efficient Compressive Image Coding for Green Internet of Things (IoT)

**DOI:** 10.3390/s18041231

**Published:** 2018-04-17

**Authors:** Ran Li, Xiaomeng Duan, Xu Li, Wei He, Yanling Li

**Affiliations:** School of Computer and Information Technology, Xinyang Normal University, Xinyang 464000, China; dxmLily@163.com (X.D.); LiXusolar@163.com (X.L.); violahewei@gmail.com (W.H.); liyanling@xynu.edu.cn (Y.L.)

**Keywords:** Green IoT, compressive sensing, image coding, gradient field, linear projection

## Abstract

Aimed at a low-energy consumption of Green Internet of Things (IoT), this paper presents an energy-efficient compressive image coding scheme, which provides compressive encoder and real-time decoder according to Compressive Sensing (CS) theory. The compressive encoder adaptively measures each image block based on the block-based gradient field, which models the distribution of block sparse degree, and the real-time decoder linearly reconstructs each image block through a projection matrix, which is learned by Minimum Mean Square Error (MMSE) criterion. Both the encoder and decoder have a low computational complexity, so that they only consume a small amount of energy. Experimental results show that the proposed scheme not only has a low encoding and decoding complexity when compared with traditional methods, but it also provides good objective and subjective reconstruction qualities. In particular, it presents better time-distortion performance than JPEG. Therefore, the proposed compressive image coding is a potential energy-efficient scheme for Green IoT.

## 1. Introduction

### 1.1. Motivation and Objective

With the proliferation of Internet of Things (IoT), we have witnessed more and more devices become interconnected over the recent years. There are many applications in the IoT framework, e.g., environmental monitoring, surveillance, device tracing, et al. One benefit from the framework of IoT is that a large amount of data can be gathered in a central processing server, so that we can analyze the data and achieve the valuable information in real time. The data transmission between devices consumes lots of energy, so a Green IoT has become the focus of both the academia and industry [1,2,3]. Since visual sensors are a major energy consumer on IoT, many existing works have made great efforts to design energy-efficient ones, e.g., CITRIC [4], MicrelEye [5]. These visual sensors are only the size of a coin, and their battery can last as long as a dozen hours. Some cooperative mechanisms [6,7] are deployed in visual sensors to reduce energy consumption by only transmitting some valuable visual features, and some energy-aware protocols are also used to save energy [8,9]. In spite of that, image coding is still a heavy burden for visual sensors, e.g., the processing part, which often uses JPEG [10] to compress images, is the most consuming component in MicrelEye. Therefore, a challenge that we face for Green IoT is to design an energy-efficient image coding.

For Green IoT, Compressive Sensing (CS) [11,12] can provide a potential scheme to compress images in visual sensor. As a unique advantage of CS, random measurement captures and represents compressible images at a rate that is significantly below the Nyquist rate, bringing about a low-energy encoder. More importantly, the less investment of energy does not affect the compression efficiency. Like traditional image encoders, the CS-base one also provides a low-bandwidth bit stream. However, the price of saving energy is a big computational burden when decoding an image. The architecture of IoT caters to the imbalance between encoder and decoder for CS-based image coding. The cloud on IoT has the sufficient ability to cope with the high-cost decoding task, but we still expect the green decoder to consider both energy saving and real-time display. Therefore, the objective of this paper is to design a CS-based image codec for Green IoT, which has both a light encoder and a light decoder while providing a good recovery quality.

### 1.2. Related Work

Lots of existing works have tried to improve the unsatisfactory rate-distortion performance of CS-based image codec. Regardless of the computational cost, these works use many complex prior models, e.g., collaborative sparsity [13], auto-regression [14], low rank [15], etc., in order to design a variety of recovery algorithms. However, with an energy-constrained CS encoder, it is difficult to significantly improve recovery quality by investing plenty of computations, because the prior model cannot always reveal the image statistics. A shortcut is to learn some information on image statistics in advance. CS theory indicates that the required number of CS measurement by perfect recovery is related to the image sparsity. Suppose that *M* is CS measurement number and *K* is the sparse degree of image. Based on experience, a satisfactory quality can be obtained only when *M* is roughly equal to 4*K* [11], so that we can use the known information on image sparsity to properly allocate measuring resources. By learning the information on image sparsity, the recovery quality can be improved significantly at the cost of a few computations. Block CS (BCS) framework [16] is suitable for performing the above scheme. The local areas in image have different waveforms, e.g., some areas have lots of smooth components, and some have strong edges, so different image blocks have different sparse degrees in a fixed space. There is no unique formula to define the sparse degree of a block, and the existing works generally use image features to measure it, e.g., DCT coefficients [17], variance [18], saliency [19], etc. Different methods to measure sparse degree bring about different recovery qualities. With an energy-constrained CS encoder, we still need a lighter method to measure the sparse degree. The non-uniform allocation of CS measurements introduces another advantage, i.e., a light decoder, which can even enable us to abandon the traditional CS recovery algorithm and turn to a linear operator, e.g., Minimum Mean Square Error (MMSE) [20], in order to decode image in real time. To conclude, our objective can be achieved by designing the measuring allocation based on sparse degree under the BCS framework.

### 1.3. Main Contribution

First, we present an energy-efficient compressive encoder, in which CS measurements are allocated based on the sparse degree of each image block. Different from the existing works, we define a block-based gradient field to measure the sparse degree. The block-based gradient field models the distribution of block sparse degree, and its computation has a low complexity, so that visual sensors are likely to reduce the energy consumption, while guaranteeing a good recovery quality.

Next, we present a real-time decoder, which learns a projection matrix to recover image blocks by matrix-vector product. Projection matrix learning depends on the MMSE criterion, which fuses the statistics of image block into the matrix so as to guarantee a good recovery quality. Different from traditional CS recovery algorithms, our real-time decoder has a low energy cost, so it is a suitable way to achieve Green IoT.

## 2. Proposed Compressive Image Coding

### 2.1. Framework Overview

**Figure 1:**
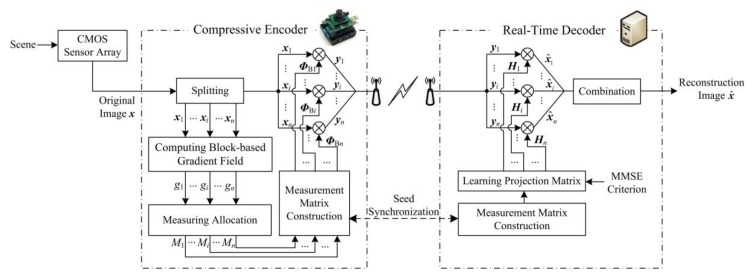
Block diagram of the proposed compressive image coding.

The block diagram of the proposed compressive image coding is depicted in Figure 1. A natural scene is captured by the CMOS sensor array to be a full-sampling image ***x*** of *N* = *I*_r_ × *I*_c_ in size. The original image ***x*** is compressed by the compressive encoder as CS measurements, which are transmitted to the real-time decoder. By using these CS measurements, the real-time decoder accurately reconstructs the estimation $\hat{x}$ of ***x***.

At the compressive encoder, the original image ***x*** is first divided into *n* blocks of *B* × *B* in size, and let ***x****_i_* represent the *i*-th block, *i* = 1, 2, …, *n*, *n* = *N*/*B*^2^. Second, we compute the block-based gradient field to measure the sparse degrees of image blocks. On the gradient field, ***x****_i_* has a gradient value *g_i_,* which reveals the relative level of sparse degree of ***x****_i_* among all of the blocks. The block-based gradient field guides the allocation of measuring resources, and it decides the measurement number *M_i_* of ***x****_i_*. Then, according to *M_i_*, we construct the measurement matrix ***Φ***_B*i*_ of ***x****_i_*. The elements in measurement matrix obey Gaussian distribution, and they are generated by a pseudorandom sequence [21]. Finally, ***x****_i_* is measured by ***Ф***_B*i*_ as CS measurement vector ***y****_i_*. All of the CS measurement vectors are transmitted to the real-time decoder.

At the real-time decoder, we first reconstruct the measurement matrices of all the blocks. These matrices are required to be the same as those at the compressive encoder. According to the property of pseudorandom sequence, we only need to synchronize the seeds of pseudorandom sequences that are used both at the encoder and at decoder. Then, depending on MMSE criterion, we learn the projection matrix ***H****_i_* of ***x****_i_*, and obtain the estimation ${\hat{x}}_{i}$ u matrix-vector product. Finally, we combine all of the block estimations into the reconstructed image $\hat{x}$. 

In the following subsections, we describe the flows of compressive encoder and real-time decoder in detail.

### 2.2. Compressive Encoder

**Figure 2:**
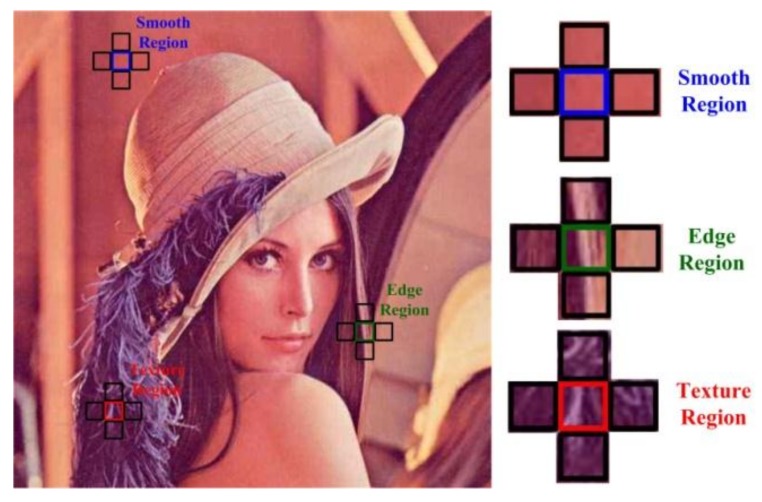
Illustration of visual contrasts among blocks on smooth, edge and texture regions of *Lenna*. Note that the size of block is 16 × 16.

The core of compressive encoder is how to measure the sparse degree of each block. As shown in Figure 2, a block that is marked in blue lies in the smooth region, it has a low sparse degree in DCT space due to its stable pixel variation, and its color is quite close to those of its neighboring blocks; a block that is marked in green lies in the edge region, it has a moderate sparse degree in DCT space due to the existence of strong edge pixels, and it exhibits a strong visual contrast in some direction; a block marked in red lies in the texture region, it has a high sparse degree in DCT space due to its unstable statistics of pixels, and there is a strong visual contrast between any two blocks. We can see from the above that the visual contrast is related to the sparse degree, so it can be regarded as an index to measure the sparse degree.

**Figure 3:**
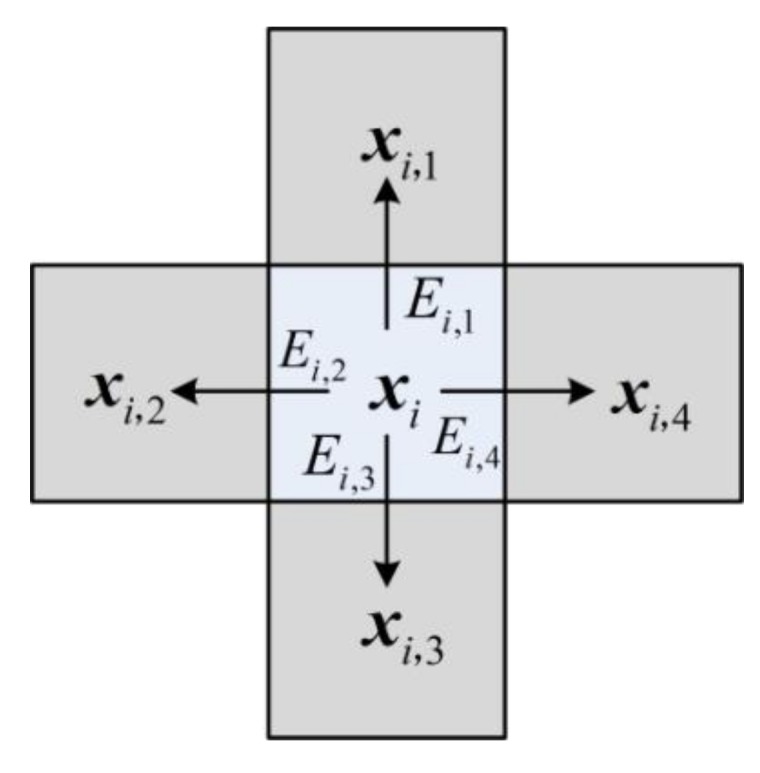
Illustration of computing the block-based gradient of ***x****_i_*.

The visual contrast can be measured by the block-based gradient, which is the maximum energy difference between the current block and its four neighboring blocks. As shown in Figure 3, we select four neighboring blocks ***x****_i_*_,*j*_ (*j* = 1, 2, 3, 4) of ***x****_i_*, and calculate the energy differences between ***x****_i_* and them, as follows,

(1) $$E_{i,j}=\frac{1}{B^{2}}{\left\|x_{i}-x_{i,j}\right\|}_{2},$$

where ||·||_2_ is the *l*_2_ norm. The block-based gradient of ***x****_i_* is defined as

(2) $$G_{i}=\max\left\{E_{i,1},E_{i,2},E_{i,3},E_{i,4}\right\},$$

where max{·} is an operator to get the maximum value from the input set. *G_i_* cannot be directly used to represent the block sparse degree, but its spatial distribution is similar to that of block degree. Therefore, we further normalize *G_i_* and generate the block-based gradient field, as follows,

(3) $$g_{i}=\frac{G_{i}}{\sum_{i=1}^{n}G_{i}},$$

where *g_i_* ranges from [0, 1]. A small *g_i_* indicates that the sparse degree of ***x****_i_* is at a low level relative to those of other blocks, and vice verse.

According to *g_i_*, we adaptively allocate the measuring resources for each block, which guarantees that more CS measurements are allocated for blocks with a high sparse degree and fewer for blocks with a low sparse degree. At first, in order to obtain the basic recovery quality, we uniformly assign 30% of the measuring resources to all of the blocks, i.e., the initial measurement number *M*_0*i*_ of ***x****_i_* is set, as follows:

(4) $$M_{0i}=0.3\times \frac{M}{n},$$

where *M* is the total measurement number of the whole image. The rest 70% of the measuring resources are adaptively allocated according to the block-based gradient field, i.e., the measurement number *M_i_* of ***x****_i_* is set, as follows,

(5) $$M_{i}=\mathrm{round}\left[0.7\cdot g_{i}\cdot M+M_{0i}\right],$$

where round[·] is rounding operator. Besides, in order to avoid the circumstance where the measurement number exceeds the total number of pixels in a block, we set an upper limit of measurement number *B*^2^ for each block, and the measurements that are exceeding the upper limit are evenly distributed to other blocks for the sake of simplicity.

After determining the measurement number *M_i_* of ***x****_i_*, we can construct the block measurement matrix ***Ф***_B*i*_, and compress ***x****_i_* by the following formula,

(6) $$y_{i}=\Phi_{Bi}\cdot x_{i},$$

in which the size of ***Φ***_B*i*_ is *M_i_* × *B*^2^, and ***y****_i_* is the CS measurement vector of length *M_i_*. The CS measurement vectors of all the blocks are packed and are transmitted to the decoder.

### 2.3. Real-Time Decoder

The real-time decoder linearly projects the received CS measurement vectors into image blocks. The projection matrix is learned according to MMSE criterion. We expect to find an estimation ${\hat{x}}_{i}$ that approaches the original block ***x****_i_* in the statistical sense, i.e., by MMSE criterion, to obtain the estimation of each block,

(7) $$\underset{{\hat{x}}_{i}}{\min}E\left[{\left\|x_{i}-{\hat{x}}_{i}\right\|}_{2}^{2}\right],$$

where ***E***[·] is the expectation function. ${\hat{x}}_{i}$ is reconstructed by the following matrix-vector product,

(8) $${\hat{x}}_{i}=H_{i}\cdot y_{i},$$

where ***H****_i_* is the projection matrix. Plug Equation (8) into the model (7), we get

(9) $$\underset{H_{i}}{\min}E\left[{\left\|x_{i}-H_{i}\cdot y_{i}\right\|}_{2}^{2}\right].$$

***y****_i_* is the measurements on ***x****_i_*, and by plugging Equation (6), the model (9) can be modified as

(10) $$\underset{H_{i}}{\min}E\left[{\left\|x_{i}-H_{i}\cdot \Phi_{Bi}\cdot x_{i}\right\|}_{2}^{2}\right]$$

Suppose that the objection of the model (10) is as follows,

(11) $$f\left[H_{i}\right]=E\left[{\left\|x_{i}-H_{i}\cdot \Phi_{Bi}\cdot x_{i}\right\|}_{2}^{2}\right].$$

The above function is convex and differentiable, so we take a derivative of *f*[***H****_i_*] with respect to ***H****_i_*, and obtain the requirement

(12) $$\frac{\partial f}{\partial H_{i}}=2H_{i}\cdot \Phi_{Bi}\cdot E\left[x_{i}x_{i}^{T}\right]\cdot \Phi_{Bi}^{T}-2E\left[x_{i}x_{i}^{T}\right]\cdot \Phi_{Bi}^{T}.$$

**Figure 4:**
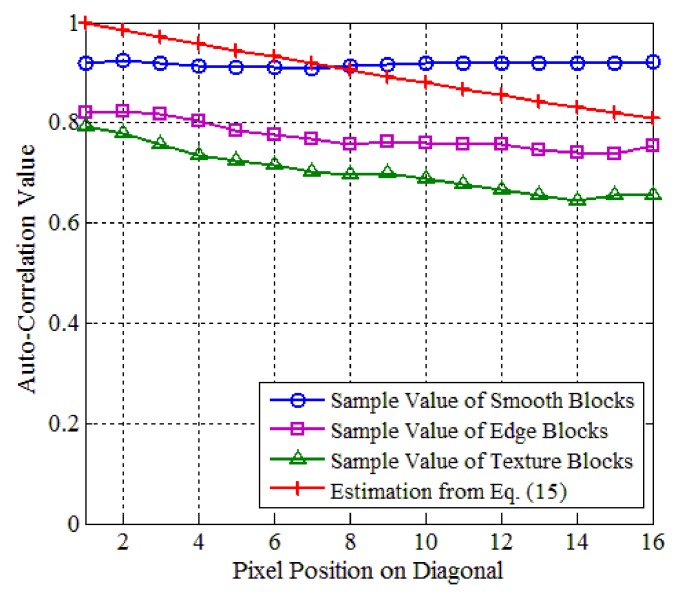
Comparison of sample and estimation of auto-correlation matrix.

By making Equation (12) to be 0, the solution of the model (10) is obtained as

(13) $$H_{i}=E\left[x_{i}x_{i}^{T}\right]\cdot \Phi_{Bi}^{T}\cdot {\left\{\Phi_{Bi}\cdot E\left[x_{i}x_{i}^{T}\right]\cdot \Phi_{Bi}^{T}\right\}}^{-1}.$$

in which *E*[***x****_i_**x**_i_*^T^] is the auto-correlation matrix of ***x****_i_*, and its constitution is as follows,

(14) $$E\left[x_{i}x_{i}^{T}\right]=\left[\begin{array}{cccc} E\left(x_{i,1}x_{i,1}\right) & E\left(x_{i,1}x_{i,2}\right) & \cdots & E\left(x_{i,1}x_{i,B^{2}}\right)\\ E\left(x_{i,2}x_{i,1}\right) & E\left(x_{i,2}x_{i,2}\right) & \cdots & E\left(x_{i,2}x_{i,B^{2}}\right)\\ \vdots & \vdots & \ddots & \vdots \\ E\left(x_{i,B^{2}}x_{i,1}\right) & E\left(x_{i,B^{2}}x_{i,2}\right) & \cdots & E\left(x_{i,B^{2}}x_{i,B^{2}}\right) \end{array}\right].$$

in which *E*[*x*_*i*,*p*_*x*_*i*,*q*_] represents the correlation between the two pixels *x*_*i*,*p*_ and *x*_*i*,*q*_ in ***x****_i_*, *p*, *q* = 1, 2, …, *B*^2^. For natural images, we approximate *E*[*x_i_*_,*p*_*x_i_*_,*q*_] using the following formulation,

(15) $$E\left[x_{i,p}x_{i,q}^{}\right]=\rho^{\delta_{p,q}},$$

where *δ_p,q_* is the chessboard distance between *x_i_*_,*p*_ and *x_i_*_,*q*_, and *ρ* is an empirical value, which is set to be 0.95. We extract smooth, edge, and texture blocks of 16 × 16 in size from 10 test images of 512 × 512 in size, and construct the sets of smooth, edge, and texture blocks, respectively. For each set, we compute the sample values of the auto-correlation matrix, and present the sample auto-correlation values of pixels on diagonal in Figure 4. From Figure 4, it can be seen that the sample values of smooth blocks vary steadily as the distance between the pixels increases, but the sample values of edge and texture blocks drop off. The estimated values of Equation (15) constitute a good fitted curve that synthesizes three different variations of smooth, edge, and texture blocks. Therefore, the estimation from Equation (15) is a good balance among the sample values of various block classes.

The linear projection method has a low computational complexity, and the whole image reconstruction only requires matrix-vector product for *n* times. Therefore, our decoder can guarantee both energy saving and real-time display.

## 3. Experimental Results

In this section, various experiments are conducted to evaluate the performance of the proposed compressive image coding. First, we evaluate the encoding complexity, and the execution time of our encoder is compared with H.264/AVC [22], HEVC [23], and DISCOVER [24], which are the traditional video coding systems. Second, the performance of our decoder is evaluated by using Peak Signal-to-Noise Ratio (PSNR) and Structural SIMilarity (SSIM) [25], and the comparison with the two popular CS recovery algorithms, OMP [26] and NESTA [27], are also presented. Finally, the performance of our overall system is compared with that of JPEG. In all of the experiments, the block size *B* is set to be 16, and we set the measurement rate *S* (=*M*/*N*) to be between 0.1 and 0.5. All of the experiments are conducted under the following computer configuration: Intel(R) Core (TM) i7 @ 3.30 GHz CPU, 8 GB, RAM, Microsoft Windows 7 64 bits, and MATLAB Version 7.6.0.324 (R2008a).

### 3.1. Encoder Evaluation

It is difficult to accurately evaluate the energy consumption that is required to encode an image, and instead, we use the execution time of encoding video sequence to indirectly reveal the energy consumption of image coding. The first 100 frames of *Foreman*, *Mobile*, *Highway*, and *Container* sequences with CIF resolution of 352 × 288 pixels are encoded, respectively, by the proposed system, H.264/AVC, HEVC, and DISCOVER, in which our system is written in MATLAB and others are programmed in C++. Our system uses CS to measure each video frame individually, and H.264/AVC, HEVC, and DISCOVER use their common configurations of inter-prediction. Note that: (1) The configurations of our encoder and these video encoders can provide a good reconstruction quality; (2) no matter it is video or image, the CS encoder remains the same, so that we can compare the encoding performance of our system with those of the traditional video encoders. Table 1 presents the time to encode 100 frames of various video coding systems. We can see that the proposed system requires much time as the measurement rate increases; however, it does not take more than 10 s even with a high measurement rate, e.g., the time to encode *Foreman* sequence requires only about 9.47 s when *S* is 0.5. H.264/AVC, HEVC, and DISCOVER take longer than that, particularly HEVC has a heavy computational burden. Although these results have a weak comparability due to the tradeoff between encoding complexity and reconstruction quality, it can be testified under the common test conditions that the encoder of the proposed scheme has a very low energy consumption when compared with these traditional video encoders.

### 3.2. Decoder Evaluation

**Figure 5:**
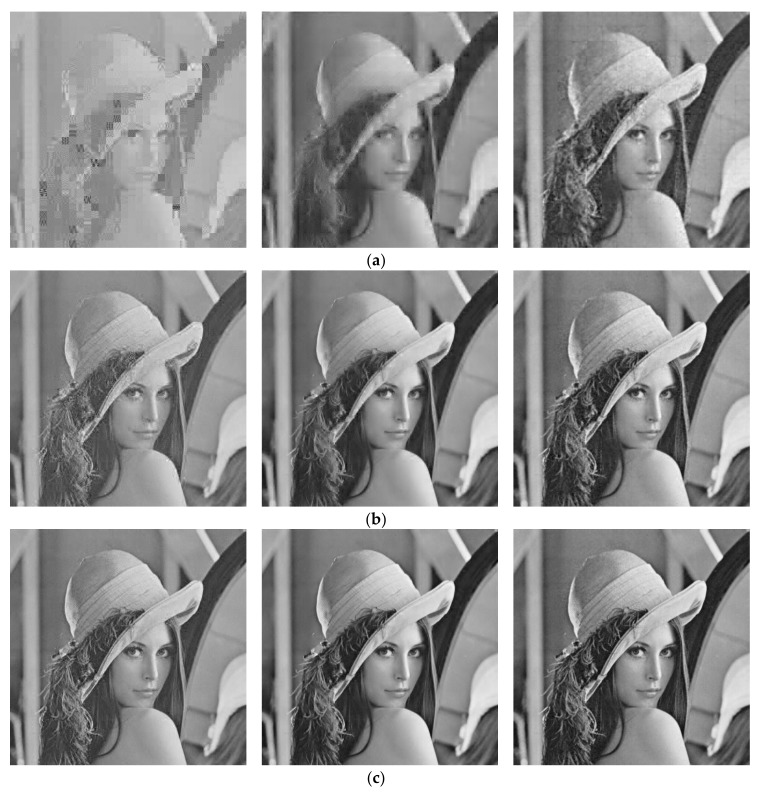
Comparison of subjective visual qualities of *Lenna* reconstructed by different recovery algorithms at *S* = 0.1, 0.3 and 0.5, respectively. From left to right: OMP, NESTA and the proposed algorithm. (**a**) *S* = 0.1, (**b**) *S* = 0.3, and (**c**) *S* = 0.5. Note that *S* is the measurement rate.

Table 2 presents PSNR and reconstruction time of our decoder, OMP and NESTA for 512 × 512 *Lenna*, *Barbara*, *Peppers*, *Goldhill,* and *Mandrill* test images when *S* is, respectively, 0.1, 0.3, and 0.5. We achieve the MATLAB source codes of OMP and NESTA from their original authors, and remain their default configurations. We can see that our real-time decoding method achieves higher PSNR values than OMP and NESTA in most cases, e.g., when *S* is 0.1, our method is 8.52 dB and 6.27 dB higher than OMP and NESTA for *Lenna*, respectively. For *Barbara*, our method is 1.11 dB lower than OMP when *S* is 0.5, and cannot provide a big PSNR gain at any measurement rate, which indicates that our method has limited a ability to recover periodic patterns. Table 2 also lists the reconstruction time of various systems. The results indicate that our system has a low computational complexity when compared with OMP and NESTA, e.g., when *S* is 0.1, our method requires only 0.88 s to recover *Lenna* while NESTA takes 198.47 s. The execution time of our system increases with the rising measurement rate, but only slightly. Table 3 presents SSIM values for test images at the measurement rate of 0.1, 0.3, and 0.5. We can see that our decoder outperforms OMP and NESRA in most cases. For *Barbara*, our decoder is 0.0944, 0.0254, and 0.0038 higher than NESTA at the measurement rate of 0.1, 0.3, and 0.5, respectively. There is still SSIM degradation for our decoder when reconstructing a few of images at a high measurement rate, e.g., when reconstructing *Mandrill* at *S* = 0.3, our decoder is 0.1346 less than NESTA, which indicates that our decoder could lose some structural information for rich-texture images, e.g., *Barbara*, *Goldhill,* and *Mandrill*, at a high measurement rate. The last column of Table 3 lists also the average SSIM values on all test images. It can be seen that our decoder achieves the better results than OMP and NESTA at *S* = 0.1 and 0.5. Figure 5 presents the visual reconstruction results of *Lenna* by various recovery algorithms at *S* = 0.1, 0.3 and 0.5, respectively. At any measurement rate, our method provides a pleasing result when compared with those that were reconstructed by OMP and NESTA. With our method, the surfaces and edges of objects are better preserved, and blocking artifacts are reduced significantly. From the above, we can see that our real-time decoding method provides better objective and subjective qualities with low energy consumption.

### 3.3. Overall Evaluation

**Figure 6:**
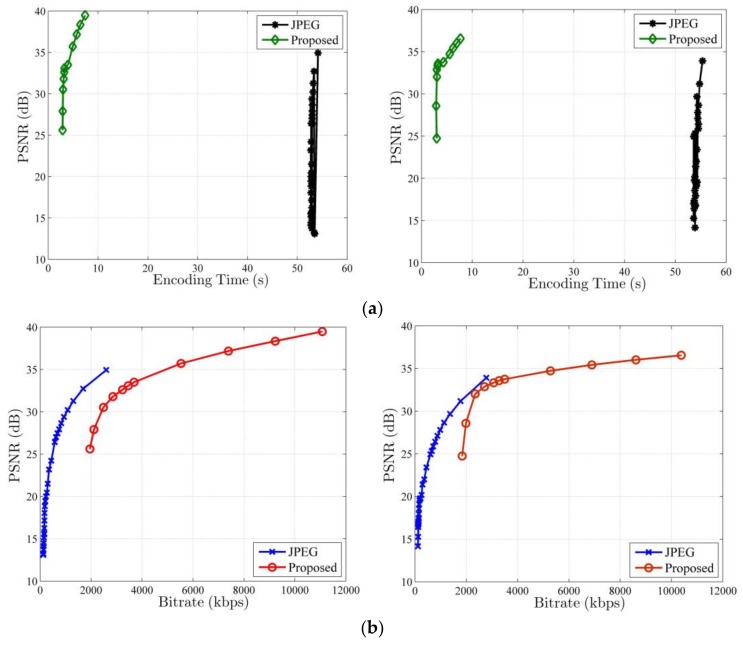
Time-distortion and rate-distortion curves of the proposed system and JEPG for *Foreman* and *Container* sequences. (**a**) Time-distortion curve and (**b**) Rate-distortion curve. Left is *Foreman*, and Right is *Container*.

Because the most of visual sensor network platforms use JPEG to compress the captured video sequence [28], we compare the performance of our overall system with JPEG, and their time-distortion and rate-distortion curves for the CIF test sequences *Foreman* and *Container* are presented in Figure 6. As shown in Figure 6a, the PSNR of our system rises rapidly as the encoding time increases, and its encoding time keeps at a low level, regardless of the PSNR values. When we set various quantization steps, the encoding time of JPEG changes little, and it is higher than that of our system. For JPEG, there is no obvious linear correlation between PSNR and encoding time, so it is difficult to control energy consumption by adjusting the reconstruction quality. From Figure 6a, we can see that our method is a superior energy-efficient image coding scheme to JPEG. However, Figure 6b shows that the rate-distortion performance of our system is not as good as that of JPEG, so we need to further improve the rate-distortion performance of our system in future.

## 4. Conclusions

In this paper, we propose an energy-efficient compressive image coding system that adaptively measures each block according to the block-based gradient field of image, which reveals the variation of block sparse degree. At the compressive encoder, under the guidance of gradient field, more CS measurements are allocated for blocks with a low sparse degree and fewer for blocks with a high sparse degree. At real-time decoder, according to MMSE criterion, a projection matrix is learned, and it is used to linearly reconstruct all of the image blocks. Experimental results show that the proposed system provides a high decoding quality with a low encoding energy. When compared with JPEG, it also shows a better time-distortion performance. However, the rate-distortion curve of our system is not so satisfactory, and in future, we will develop more efficient decoding schemes to improve the rate-distortion performance.

As the research in this paper is exploratory, there are many intriguing questions that future work should consider. First, our image coding scheme should be deployed into an actual hardware platform. By the energy consumption of our scheme on this hardware platform, we are to verify the trade-off among the number of CS measurements, energy consumption, and the reconstruction quality. Second, we should analyze the trade-off on energy between local codec and transmission. Importantly, by some simulation results, we hope to construct an empirical model to measure this trade-off in the future work.

## Figures and Tables

**Table 1:** The time to encode 100 frames of various video coding systems.

Sequence	Time/s					
	Proposed			H.264/AVC	HEVC	DISCOVER
	*S* = 0.1	*S* = 0.3	*S* = 0.5			
*Foreman*	3.91	6.54	9.47	389.41	2306.60	36.40
*Mobile*	3.93	6.49	9.44	296.32	2880.12	60.08
*Highway*	3.94	6.48	9.84	372.60	1550.31	31.21
*Container*	3.97	6.51	9.79	292.83	1301.99	40.65

**Table 2:** Peak Signal-to-Noise Ratio (PSNR) (in dB) and reconstruction time (in s) of different recovery algorithms.

Algorithm	*S*	*Lenna*		*Barbara*		*Peppers*		*Goldhill*		*Mandrill*	
		PSNR/dB	Time/s	PSNR/dB	Time/s	PSNR/dB	Time/s	PSNR/dB	Time/s	PSNR/dB	Time/s
OMP	0.1	18.89	2.80	16.64	2.79	17.28	2.80	20.49	2.81	15.71	2.81
NESTA		21.14	198.47	18.90	174.16	20.25	197.28	21.11	115.32	18.04	159.45
Proposed		27.41	0.88	21.78	0.88	26.79	0.88	26.30	0.89	19.76	0.88
OMP	0.3	27.35	4.29	24.02	4.22	24.35	5.40	23.86	4.34	17.64	4.34
NESTA		27.28	134.34	22.99	170.11	26.86	126.01	26.32	125.42	20.46	117.25
Proposed		32.67	1.77	24.68	1.66	31.36	1.70	30.40	1.68	22.91	1.65
OMP	0.5	31.64	5.97	28.35	5.97	31.11	6.08	29.19	6.07	21.04	5.97
NESTA		30.83	120.89	25.52	100.81	31.16	117.19	29.63	110.17	22.77	196.12
Proposed		36.04	2.93	27.24	2.82	34.11	2.93	33.40	2.88	25.62	2.75

**Table 3:** Structural SIMilarity (SSIM) comparison of different recovery algorithms.

Algorithm	*S*	*Lenna*	*Barbara*	*Peppers*	*Goldhill*	*Mandrill*	*Avg.*
OMP	0.1	0.5953	0.4823	0.5589	0.5314	0.3312	0.4998
NESTA		0.7211	0.6104	0.7385	0.6149	0.4322	0.6234
Proposed		0.8249	0.7048	0.8300	0.7638	0.5876	0.7422
OMP	0.3	0.8850	0.8482	0.8826	0.8207	0.6334	0.8140
NESTA		0.9036	0.8256	0.9139	0.9429	0.9596	0.9091
Proposed		0.9409	0.8510	0.9318	0.9147	0.8250	0.8927
OMP	0.5	0.9515	0.9367	0.9412	0.9164	0.7946	0.9081
NESTA		0.9581	0.9147	0.9596	0.9352	0.8548	0.9245
Proposed		0.9712	0.9185	0.9610	0.9595	0.9148	0.9450
